# Metabolic syndrome and the risk of perioperative ischemic stroke in non-cardiac surgery: a case-control retrospective study

**DOI:** 10.3389/fendo.2026.1785277

**Published:** 2026-03-11

**Authors:** Mengyao Qu, Yanan He, Lu Yu, Yingfu Li, Lei Yan, Huikai Yang, Rui Wang, Yixun Lu, Miao Sun, Hang Guo, Weidong Mi, Yulong Ma

**Affiliations:** 1 Department of Anesthesiology, The First Medical Center of Chinese PLA General Hospital, Beijing, China; 2 National Clinical Research Center for Geriatric Diseases, Chinese PLA General Hospital, Beijing, China; 3 Department of Anesthesia, People’s Hospital of Xinjiang Uygur Autonomous Region, Urumqi, China; 4 Department of Anesthesiology, The Seventh Medical Center of Chinese PLA General Hospital, Beijing, China

**Keywords:** blood pressure, glucose, metabolic syndrome, non-cardiac surgery, perioperative ischemic stroke

## Abstract

**Background:**

Perioperative ischemic stroke (PIS), although rare, is a devastating complication following surgery. Metabolic syndrome (MetS), also known as insulin resistance syndrome, is characterized by obesity, diabetes, hypertension and dyslipidemia. MetS has been reported to be associated with surgical complications. However, the association between PIS and MetS remains unclear.

**Methods:**

We conducted a case-control study by selecting 139,191 participants from 223,415 non-cardiac surgery patients at the Chinese PLA General Hospital between 2008 and 2019. Univariate and multivariate logistic regression analyses were performed to examine the association of MetS and PIS. Propensity score matching (PSM) and inverse probability of treatment weighting (IPTW) were applied to address the potential confounding effects of covariates. Subgroup and sensitivity analyses were performed to verify the robustness of the association between MetS and PIS.

**Results:**

Among 139,191 participants, 328 (0.24%) developed PIS. Participants with MetS had a 2.18-fold increased risk of PIS. In the PSM and IPTW models, the odds ratios for MetS were 1.41 and 1.35, respectively. Subgroup analyses showed that the association remained significant. In sensitivity analyses, the results remained robust after excluding patients on specific medications and those with a prognostic nutrition index (PNI) less than 38.8.

**Conclusions:**

This study confirms MetS as an independent predictor of PIS among non-cardiac surgical patients in China, with elevated blood pressure and glucose as the principal drivers of this association. Abnormal parameters in MetS, especially elevated glucose levels and elevated blood pressure, are significantly associated with increased odds of PIS. Surgical patients with MetS require increased attention to PIS prevention.

## Introduction

Stroke is the second leading cause of mortality globally, with projections indicating a 50% increase in stroke-related deaths by 2050 (1). Annually, approximately 300 million adults worldwide undergo non-cardiac surgery (2, 3). Perioperative ischemic stroke (PIS), although relatively uncommon, is a potentially devastating condition, with over 600,000 surgical patients affected globally each year (4). Epidemiological evidence suggests that more than 50% of patients who survive a perioperative stroke will experience severe disability and be discharged to a location other than home (5–7). Additionally, more than 20% of patients with an acute perioperative stroke will die within 30 days of surgery (5–7). Due to delayed recognition, infrequent intervention, and high rates of disability and mortality, it is urgent to improve clinical practices for the prevention and treatment of PIS (3).

Several risk factors associated with PIS have been identified, including age, type of surgical procedure, anesthetic techniques, presence of atrial fibrillation, perioperative hemodynamics and exposure to particulate matter pollution (4). In recent years, we extracted perioperative data from 376,933 patients and established a PIS database including 223,415 patients treated between January 2008 and August 2019 at the Chinese People’s Liberation Army (PLA) General Hospital. Based on this database, we found that elevated systemic-immune inflammation index (SII), hyperglycemia, reduced prognostic nutritional index (PNI), high body mass index (BMI) and coronary heart disease (CHD) are associated with an increased risk of PIS (8–12). However, further investigation into other potential risk factors is required, and scientific management and clinical efforts are needed to advance PIS prevention.

Metabolic syndrome (MetS) is characterized by a cluster of abnormalities defined by the presence of three or more of its five components: elevated waist circumference, elevated glucose level, elevated blood pressure, elevated triglycerides (TG), and reduced high-density lipoprotein cholesterol (HDL-C) (13, 14). The prevalence varies from 12% to 37% in Asians and 12% to 26% in Europeans and is rising year by year (13, 14). Each of the metabolic components, individually or collectively, can be risk factors for cardiovascular events, stroke recurrence and all-cause mortality (15, 16). Studies have indicated a relationship between MetS and various surgical complications, including surgical site infection, postoperative delirium, respiratory complications, renal complications, cardiovascular complications, deep vein thrombosis and readmission (17–21). It has also been reported that people with MetS have a significantly higher risk of ischemic stroke than those without MetS (22). However, while a recent large-scale meta-analysis by Norris et al. (31) confirmed that MetS is associated with increased stroke risk across multiple surgical specialties, the specific association between MetS and PIS in large non-cardiac surgical cohorts, particularly in Chinese populations with distinct metabolic profiles, and the relative contribution of individual MetS components to this risk, remain insufficiently characterized.

Therefore, based on our PIS database, we systematically conducted this retrospective case-control study. This large sample research encompasses 139,191 non-cardiac patients, including 29,816 with MetS. The study aims to elucidate the potential relationship between MetS and the risk of PIS and to explore new clinical targets for PIS prevention.

## Methods

**Figure 1:**
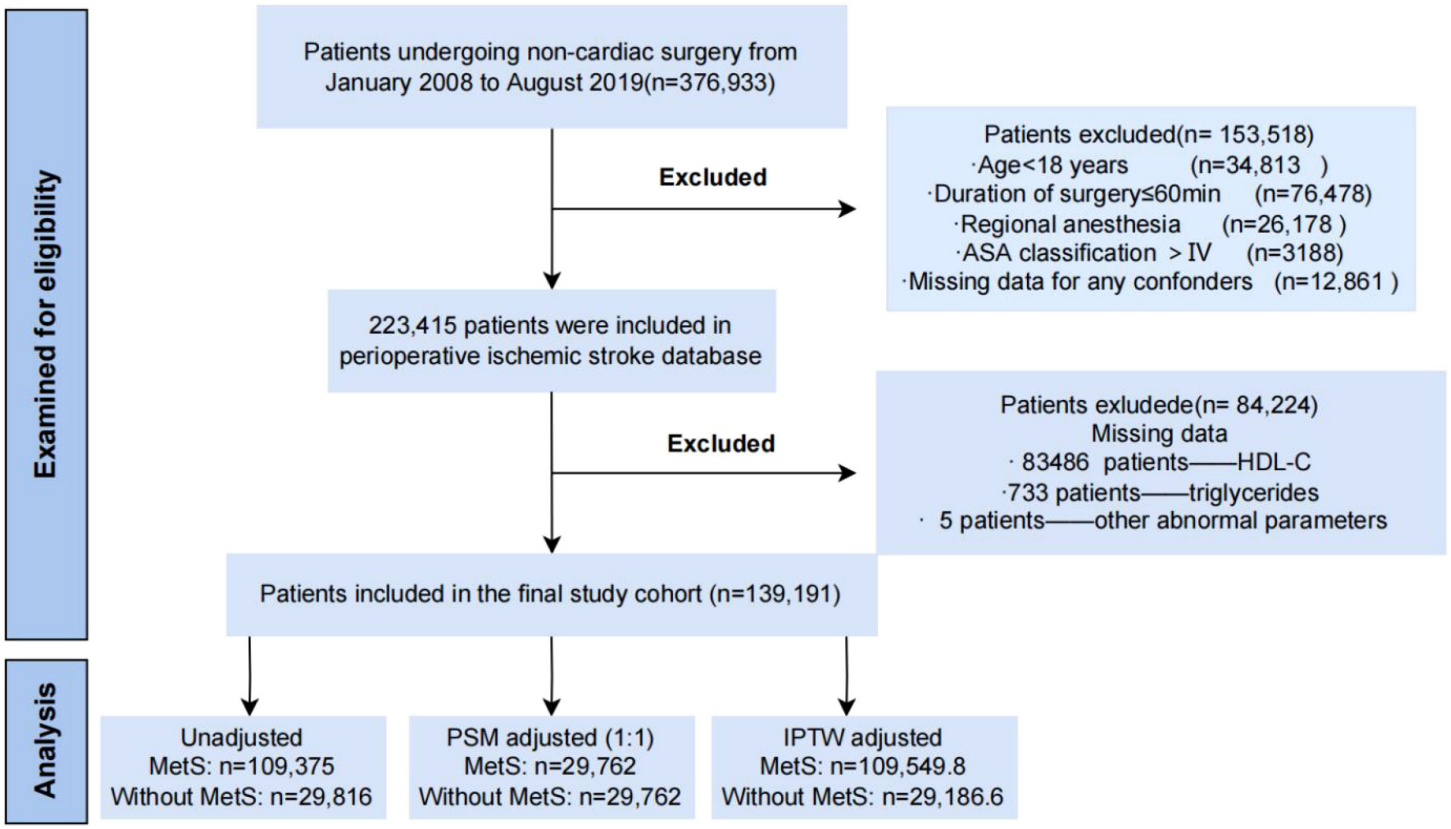
Study flow diagram highlighting patient inclusion and exclusion criteria.

**Figure 2:**
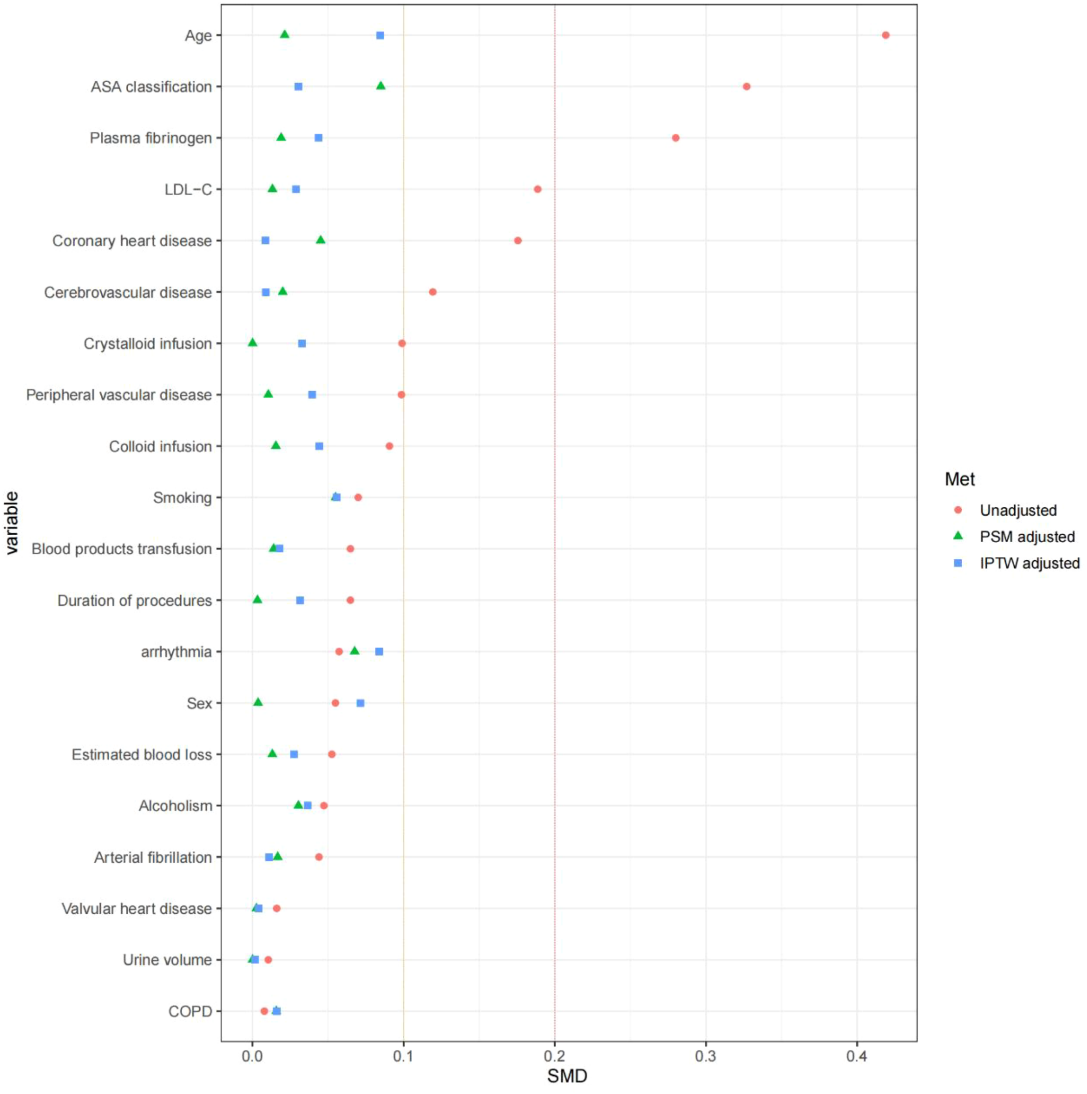
Covariate balance assessment. SMD, standardized mean differences; PSM, propensity score full matching; IPTW, inverse probability of treatment weighting.

**Figure 3:**
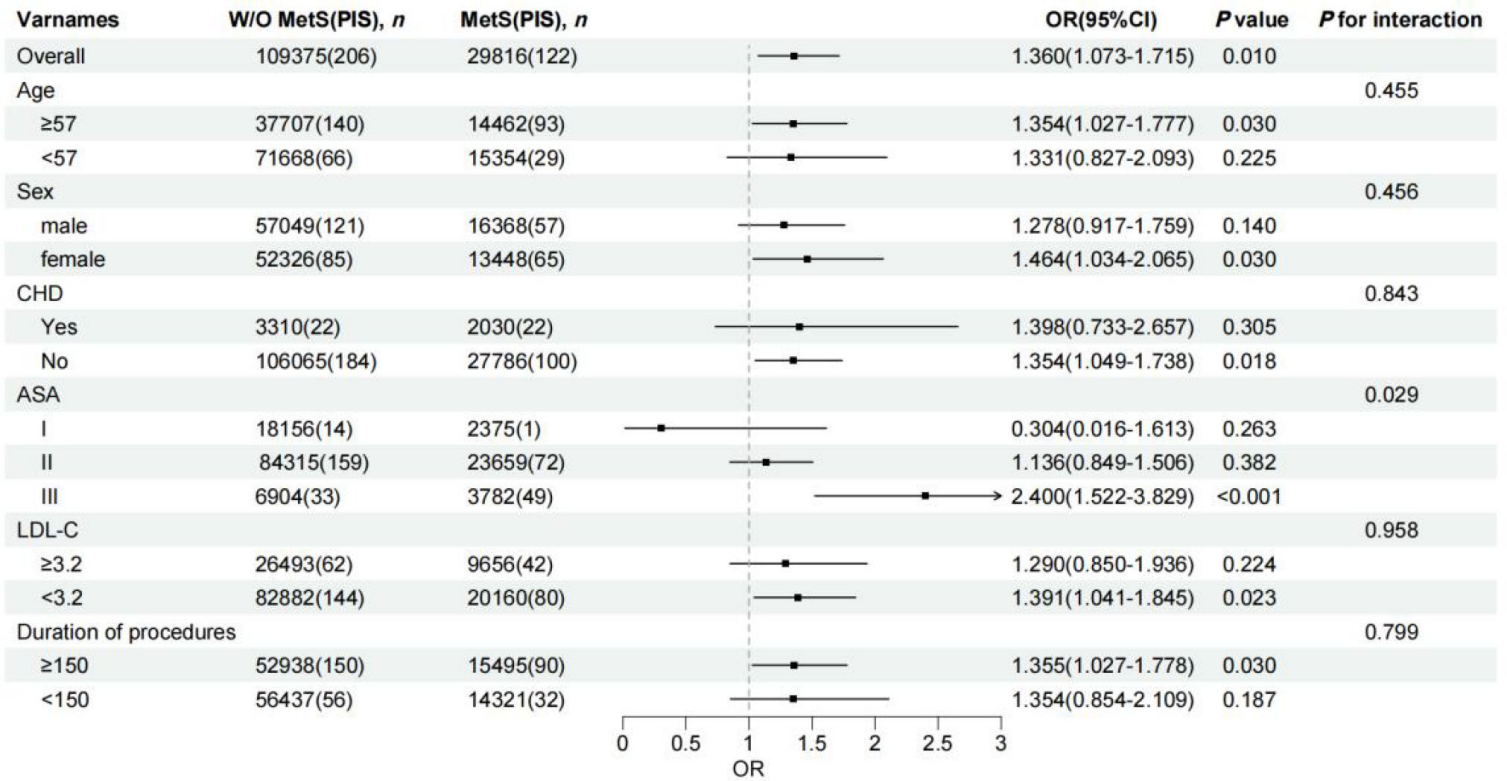
Forest plots of odds ratio for the perioperative ischemic stroke in different subgroups. OR, odds ratio; CI, confidence interval; MetS, metabolic syndrome; PIS, perioperative ischemic stroke; CHD, coronary heart disease; LDL-C, low density lipoprotein cholesterol.

The study protocol was reviewed and approved by the Institutional Ethics Committee of the Chinese PLA General Hospital (NO. S2024-518-01), and the need for informed consent was waived, due to the retrospective use of clinical records. This manuscript adheres to the applicable guidelines as presented in the Strengthening the Reporting of Observational Studies in Epidemiology (STROBE) (**Table 1**). The **Supplementary Material** include: **Table 1** (STROBE reporting checklist); **Table 2** (ICD codes used to identify PIS); **Figure 1** (directed acyclic graph illustrating causal assumptions); **Figure 2** (kernel density plots and balance diagnostics before and after PSM and IPTW); **Table 3** (full univariate logistic regression results); **Table 4** (sensitivity analysis excluding patients on cardiocerebrovascular medications); **Table 5** (sensitivity analysis excluding patients with low PNI); **Figure 3** (correlation matrix of risk factors and MetS components); and **Supplementary Table 6** (pairwise correlation coefficients among all variables).

**Table 1:** Definition of metabolic syndrome.

Component	Standard criteria	Criteria used in present study
Elevated waist circumference	Population- and country-specific definitions	BMI ≥ 30 kg/m2
Elevated Triglycerides	TG ≥ 150 mg/dL (1.7 mM), or drug treatment	Fasting TG ≥ 150 mg/dL (1.7 mM)
Reduced HDL-C	HDL-C < 40 mg/dL (1.0 mM) in males; HDL-C < 50 mg/dL (1.3 mM) in females; or drug treatment	HDL-C < 40 mg/dL (1.0 mM) in males; HDL-C < 50 mg/dL (1.3 mM) in females
Elevated blood pressure	Systolic ≥ 130 and/or diastolic ≥ 85mmHg, or drug treatment	Systolic ≥ 130 and/or diastolic≥ 85 mmHg, or drug treatment
Elevated glucose	≥ 100 mg/dL in plasma, or drug treatment	1. Fasting blood glucose ≥ 100 mg/dL (5.5 mM) and/or 2. Diabetes based on self-report and/or local hospital records

*The metabolic syndrome is defined as the presence of at least 3 of the 5 components.

**Table 2:** Key baseline clinical characteristics of unadjusted, propensity score-matched, inverse probability of treatment-weighted patients with stratification by metabolic syndrome.

Characteristics	Unadjusted patients (N = 139,191)				PSM adjusted (1:1) (N = 59,524)				IPTW adjusted (N = 138,736.4)			
	Patients without MetS (N = 109,375)	Patients with MetS (N = 29,816)	*p*	SMD	Patients without MetS (N = 29,762)	Patients with MetS (N = 29,762)	*p*	SMD	Patients without MetS (N = 109549.8)	Patients with MetS (N = 29186.6)	*p*	SMD
Demographics												
PIS, n (%)	206 (0.2)	122 (0.4)	<.001	0.04	79 (0.3)	118 (0.4)	0.007	0.023	233.7 (0.2)	85.5 (0.3)	0.007	0.016
Age, years (median [IQR])	50 (40,61)	56 (48,64)	<.001	0.419	56 (47,65)	56 (48,64)	0.001	0.021	52 (41,62)	53 (44,61)	<.001	0.085
Male sex, n (%)	57049 (52.2)	16368 (54.9)	<.001	0.055	16288 (54.7)	16343 (54.9)	0.657	0.004	58127.5 (53.1)	16521.2 (56.6)	<.001	0.071
Smoking, n (%)			<.001	0.07			<.001	0.055			<.001	0.056
never	93319 (85.3)	24689 (82.8)			24502 (82.3)	24649 (82.8)			92763.7 (84.7)	24376.0 (83.5)		
ever	5555 (5.1)	1696 (5.7)			2061 (6.9)	1689 (5.7)			6014.1 (5.5)	1462.6 (5.0)		
now	10501 (9.6)	3431 (11.5)			3199 (10.7)	3424 (11.5)			10772.0 (9.8)	3347.9 (11.5)		
Alcoholism, n (%)			<.001	0.047			0.001	0.03			<.001	0.037
never	91415 (83.6)	24389 (81.8)			24210 (81.3)	24346 (81.8)			91025.2 (83.1)	23918 (81.9)		
ever	4586 (4.2)	1410 (4.7)			1603 (5.4)	1406 (4.7)			4875.4 (4.5)	1271.5 (4.4)		
now	13374 (12.2)	4017 (13.5)			3949 (13.3)	4010 (13.5)			13649.2 (12.5)	3996.8 (13.7)		
Previous medical history												
Coronary heart disease, n (%)	3310 (3)	2030 (6.8)	<.001	0.176	1663 (5.6)	1985 (6.7)	<.001	0.045	4256.1 (3.9)	1182.1 (4.1)	0.145	0.008
Arterial fibrillation, n (%)	331 (0.3)	178 (0.6)	<.001	0.044	139 (0.5)	175 (0.6)	0.048	0.017	390.4 (0.4)	124.0 (0.4)	0.066	0.011
Arrhythmia, n (%)	18257 (16.7)	4356 (14.6)	<.001	0.057	5079 (17.1)	4345 (14.6)	<.001	0.068	18465.6 (16.9)	4039.2 (13.8)	<.001	0.084
Cerebrovascular disease, n (%)	2702 (2.5)	1396 (4.7)	<.001	0.119	1244 (4.2)	1366 (4.6)	0.015	0.02	3268.9 (3.0)	916.4 (3.1)	0.132	0.009
TIA, n (%)	2596 (2.4)	1334 (4.5)	<.001	0.116	1182 (4.0)	1307 (4.4)	0.011	0.021	3130.5 (2.9)	894.1 (3.1)	0.046	0.012
Ischemic stroke, n (%)	2347 (2.1)	1228 (4.1)	<.001	0.113	1106 (3.7)	1201 (4.0)	0.046	0.017	2869.9 (2.6)	795.7 (2.7)	0.27	0.007
Valvular heart disease, n (%)	355 (0.3)	126 (0.4)	0.012	0.016	121 (0.4)	126 (0.4)	0.799	0.003	380.2 (0.3)	108.8 (0.4)	0.51	0.004
Peripheral vascular disease, n (%)	3927 (3.6)	1687 (5.7)	<.001	0.099	1593 (5.4)	1664 (5.6)	0.207	0.01	4275.4 (3.9)	1372.4 (4.7)	<.001	0.039
COPD, n (%)	847 (0.8)	252 (0.8)	0.235	0.008	297 (1.0)	252 (0.8)	0.059	0.016	923.5 (0.8)	204.7 (0.7)	0.015	0.016
Hypertension, n (%)	16835 (15.4)	11615 (39)	<.001	0.549	6181 (20.8)	11575 (38.9)	<.001	0.404	18339.3 (16.7)	9988.0 (34.2)	<.001	0.409
Diabetes, n (%)	7993 (7.3)	9778 (32.8)	<.001	0.671	2752 (9.2)	9747 (32.7)	<.001	0.603	8532.9 (7.8)	8702.1 (29.8)	<.001	0.588
Lipid-lowering medication, n (%)	1920 (1.8)	1624 (5.4)	<.001	0.199	791 (2.7)	1607 (5.4)	<.001	0.14	2234.9 (2.0)	1275.5 (4.4)	<.001	0.133
Anticoagulant medication, n (%)	3154 (2.9)	1520 (5.1)	<.001	0.113	1110 (3.7)	1501 (5.0)	<.001	0.064	3453.6 (3.2)	1189.1 (4.1)	<.001	0.049
Hypoglycemic medication, n (%)	2445 (2.2)	3840 (12.9)	<.001	0.411	922 (3.1)	3827 (12.9)	<.001	0.366	2675.5 (2.4)	3312.1 (11.3)	<.001	0.357
ACEI medication, n (%)	1854 (1.7)	1421 (4.8)	<.001	0.174	630 (2.1)	1414 (4.8)	<.001	0.145	1998.8 (1.8)	1241.8 (4.3)	<.001	0.142
ARB medication, n (%)	3136 (2.9)	2906 (9.7)	<.001	0.286	1149 (3.9)	2894 (9.7)	<.001	0.235	3403.0 (3.1)	2541.8 (8.7)	<.001	0.239
β-blockers medication, n (%)	3050 (2.8)	2541 (8.5)	<.001	0.25	1171 (3.9)	2520 (8.5)	<.001	0.189	3411.8 (3.1)	2093.5 (7.2)	<.001	0.185
Aspirin medication, n (%)	3588 (3.3)	2067 (6.9)	<.001	0.166	1480 (5.0)	2043 (6.9)	<.001	0.08	4112.6 (3.8)	1537.2 (5.3)	<.001	0.073
Butylphthalide medication, n (%)	142 (0.1)	82 (0.3)	<.001	0.032	44 (0.1)	80 (0.3)	0.002	0.027	157.6 (0.1)	65.2 (0.2)	0.003	0.019
Edaravone medication, n (%)	238 (0.2)	118 (0.4)	<.001	0.032	63 (0.2)	118 (0.4)	<.001	0.034	241.2 (0.2)	107.3 (0.4)	<.001	0.027
Dextran medication, n (%)	401 (0.4)	166 (0.6)	<.001	0.028	137 (0.5)	166 (0.6)	0.107	0.014	416.9 (0.4)	134.7 (0.5)	0.044	0.013
Surgical related factors												
ASA classification, n (%)			<.001	0.327			<.001	0.085			<.001	0.03
I	18156 (16.6)	2375 (8)			1862 (6.3)	2375 (8.0)			16114.1 (14.7)	4002.4 (13.7)		
II	84315 (77.1)	23659 (79.4)			24596 (82.6)	23651 (79.5)			84932.2 (77.5)	22811.6 (78.2)		
III	6904 (6.3)	3782 (12.7)			3304 (11.1)	3736 (12.6)			8503.5 (7.8)	2372.6 (8.1)		
Surgical procedures, n (%)			<.001	0.227			<.001	0.157			<.001	0.163
ENT	15554 (14.2)	2915 (9.8)			3273 (11.0)	2913 (9.8)			14897.1 (13.6)	3348.3 (11.5)		
Trauma surgery	1264 (1.2)	342 (1.1)			317 (1.1)	338 (1.1)			1245.9 (1.1)	332.4 (1.1)		
Obstetrics	3753 (3.4)	1064 (3.6)			947 (3.2)	1064 (3.6)			3665.6 (3.3)	1067.2 (3.7)		
Intra-abdominal surgery	20869 (19.1)	5919 (19.9)			6965 (23.4)	5901 (19.8)			21875.8 (20.0)	5149.1 (17.6)		
Joint arthroplasty	4487 (4.1)	1738 (5.8)			1373 (4.6)	1737 (5.8)			4600.9 (4.2)	1505.1 (5.2)		
Spine	9112 (8.3)	3323 (11.1)			2600 (8.7)	3320 (11.2)			9192.8 (8.4)	3227.6 (11.1)		
Stomatology	7285 (6.7)	1473 (4.9)			1806 (6.1)	1470 (4.9)			7194.6 (6.6)	1515.6 (5.2)		
Urologic surgery	12635 (11.6)	4324 (14.5)			3731 (12.5)	4319 (14.5)			12942.5 (11.8)	4154.5 (14.2)		
General surgery	9548 (8.7)	2136 (7.2)			2146 (7.2)	2136 (7.2)			9137.8 (8.3)	2360.0 (8.1)		
Other (plastic surgery, etc)	2766 (2.5)	567 (1.9)			600 (2.0)	567 (1.9)			2656.4 (2.4)	597.9 (2.0)		
Neurosurgery	14452 (13.2)	3726 (12.5)			3407 (11.4)	3722 (12.5)			14029.9 (12.8)	3984.5 (13.7)		
Thoracic	6348 (5.8)	1736 (5.8)			2048 (6.9)	1733 (5.8)			6652.4 (6.1)	1527.4 (5.2)		
Vascular	1302 (1.2)	553 (1.9)			549 (1.8)	542 (1.8)			1458.1 (1.3)	417.0 (1.4)		
Dosage of sufentanil, ug, (median [IQR])	25 (0,45)	25 (0,50)	<.001	0.039	30 (0, 50)	25 (0, 50)	<.001	0.038	30 (0, 45)	25 (0, 45)	0.148	0.007
Duration of procedures, min, (median [IQR])	145 (100,210)	152 (105,220)	<.001	0.065	153.00 (105, 220)	152.00 (105, 220)	0.864	0.003	146 (100, 211)	150 (102, 217)	<.001	0.031
Duration of MAP<50mmHg, min, (median [IQR])	0 (0,10)	0 (0,10)	<.001	0.028	0 (0, 10)	0 (0, 10)	0.032	0.021	0 (0, 10)	0 (0, 10)	<.001	0.024
Use of blood products, n (%)	10204 (9.3)	3369 (11.3)	<.001	0.065	3223 (10.8)	3354 (11.3)	0.089	0.014	10569.3 (9.6)	2971.7 (10.2)	0.007	0.018
Estimated blood loss, mL, (median [IQR])	100 (30,200)	100 (50,200)	<.001	0.053	100 (50, 200)	100 (50., 200)	0.015	0.013	100 (31, 200)	100 (50, 200)	<.001	0.027
Urine volume, mL, (median [IQR])	254.733 (100,600)	300 (100,600)	<.001	0.01	300 (100, 600)	300 (100, 600)	0.993	<.001	265.25 (100, 600)	253.63 (100, 600)	0.501	0.002
Colloid volume, mL, (median [IQR])	500 (0,500)	500 (0,1000)	<.001	0.091	500 (0, 1000)	500 (0, 1000)	0.184	0.015	500 (0, 500)	500 (0, 1000)	<.001	0.044
Crystalloid volume, mL, (median [IQR])	1350 (1000,2000)	1500 (1100,2100)	<.001	0.099	1500 (1100, 2100)	1500 (1100, 2100)	0.869	<.001	1500 (1000, 2000)	1500 (1000, 2050)	<.001	0.033
Preoperative laboratory data												
Fibrinogen, g/L, (median [IQR])	2.94 (2.5,3.5)	3.2 (2.74,3.8)	<.001	0.28	3.15 (2.68, 3.79)	3.20 (2.74, 3.80)	<.001	0.019	2.98 (2.53, 3.57)	3.07 (2.63, 3.62)	<.001	0.044
LDL-C, mmol/L, (median [IQR])	2.65 (2.17,3.18)	2.83 (2.3,3.39)	<.001	0.189	2.82 (2.32, 3.37)	2.83 (2.30, 3.39)	0.805	0.013	2.69 (2.20, 3.23)	2.74 (2.22, 3.28)	<.001	0.029
HDL-C, mmol/L, (median [IQR])	1.2 (1.02,1.43)	0.92 (0.8,1.07)	<.001	1.051	1.19 (1.01, 1.42)	0.92 (0.80, 1.07)	<.001	1.023	1.20 (1.01, 1.42)	0.92 (0.79, 1.06)	<.001	1.084
Reduced HDL, n (%)	41328 (37.8)	26589 (89.2)	<.001	1.262	11027 (37.1)	26542 (89.2)	<.001	1.284	41039.2 (37.5)	26216.3 (89.8)	<.001	1.298
Triglyceride, mmol/L, (median [IQR])	1.09 (0.81,1.45)	2.02 (1.59,2.68)	<.001	1.029	1.12 (0.85, 1.47)	2.02 (1.59, 2.68)	<.001	1.015	1.10 (0.81, 1.45)	2.06 (1.66, 2.76)	<.001	1.046
Elevated triglyceride, n (%)	15272 (14)	21457 (72)	<.001	1.446	4206 (14.1)	21431 (72.0)	<.001	1.44	15260.0 (13.9)	21620.1 (74.1)	<.001	1.523
Glucose, mM, (median [IQR])	4.72 (4.41,5.1)	5.56 (4.84,6.43)	<.001	0.726	4.79 (4.46, 5.19)	5.56 (4.84, 6.42)	<.001	0.636	4.74 (4.42, 5.12)	5.50 (4.79, 6.30)	<.001	0.655
Elevated fasting glucose, n (%)	15970 (14.6)	18938 (63.5)	<.001	1.159	5295 (17.8)	18887 (63.5)	<.001	1.05	16872.4 (15.4)	17577.9 (60.2)		1.042
BMI, kg·m^-2^, (median [IQR])	23.739 (21.484,25.991)	26.644 (24.221,29.7)	<.001	0.881	24.01 (21.80, 26.17)	26.64 (24.22, 29.70)	<.001	0.835	23.83 (21.54, 26.03)	26.81 (24.39, 30.04)	<.001	0.913
Underweight (BMI<18.5 kg·m^-2^), n (%)	5479 (5)	223 (0.7)	<.001	0.257	1289 (4.3)	220 (0.7)	<.001	0.23	5361.7 (4.9)	212.3 (0.7)	<.001	0.254
Normal weight (18.5-24.9 kg·m^-2^), n (%)	65358 (59.8)	9490 (31.8)	<.001	0.584	17403 (58.5)	9461 (31.8)	<.001	0.557	65152.6 (59.5)	8876.6 (30.4)	<.001	0.611
Overweight (25-29.9 kg·m^-2^), n (%)	35624 (32.6)	13027 (43.7)	<.001	0.23	10297 (34.6)	13011 (43.7)	<.001	0.188	36121.5 (33.0)	12690.0 (43.5)	<.001	0.217
Obesity (≥30kg·m^-2^), n (%)	2914 (2.7)	7076 (23.7)	<.001	0.655	773 (2.6)	7070 (23.8)	<.001	0.659	2914.0 (2.7)	7407.7 (25.4)	<.001	0.693
Elevated BP, n (%)	40650 (37.2)	25231 (84.6)	<.001	1.113	13338 (44.8)	25182 (84.6)	<.001	0.916	42601.7 (38.9)	24080.3 (82.5)	<.001	0.998
Number of MetS components, n (%)			<.001	3.283			<.001	3.551			<.001	3.355
0	29161 (26.7)	0			6375 (21.4)	0 (0.0)			28011.8 (25.6)	0.0 (0.0)		
1	44294 (40.5)	0			12135 (40.8)	0 (0.0)			44388.8 (40.5)	0.0 (0.0)		
2	35920 (32.8)	0			11252 (37.8)	0 (0.0)			37149.2 (33.9)	0.0 (0.0)		
3	0	21060 (70.6)			0 (0.0)	21021 (70.6)			0.0 (0.0)	20885.5 (71.6)		
4	0	7669 (25.7)			0 (0.0)	7656 (25.7)			0.0 (0.0)	7259.7 (24.9)		
5	0	1087 (3.6)			0 (0.0)	1085 (3.6)			0.0 (0.0)	1041.4 (3.6)		

MetS, metabolic syndrome; PIS, perioperative ischemic stroke; TIA, transient ischemic attack; COPD, chronic obstructive pulmonary disease; ACEI, angiotensin converting enzyme inhibitor; ARB, angiotensin receptor blocker; ASA, American Society of Anesthesiologists; ENT, ear, nose and throat; MAP, mean arterial pressure; LDL-C, low density lipoprotein cholesterol; HDL-C, high density lipoprotein cholesterol; BMI, body mass index, BP, blood pressure.

**Table 3:** Risk of perioperative ischemic stroke according to metabolic parameters.

BMI						
		Cut-point	n with PIS/N total	Model 1	Model 2	Model 3
Quartiles	1	11.34-22.03	65/34752	Ref	Ref	Ref
	2	22.03-24.24	85/34696	1.11(0.8,1.55)	1.33(0.97,1.85)	1.13(0.81,1.57)
	3	24.24-26.78	89/35126	1.08(0.78,1.5)	1.38(1,1.91)	1.1(0.79,1.53)
	4	26.78-53.91	89/34617	1.12(0.81,1.56)	1.41(1.02,1.95)	1.14(0.83,1.59)
	*P_trend_*			0.561	0.044	0.493
	OR (95% CI) per unit increment			1.01(0.98,1.04)	1.03(1,1.06)	1.01(0.98,1.04)
	*P*-value			0.583	0.061	0.505
TG						
		Cut-point	n with PIS/N total	Model 1	Model 2	Model 3
Quartiles	1	0-2.066	73/34418	Ref	Ref	Ref
	2	2.066-3.003	76/34779	0.77(0.56,1.07)	1(0.72,1.38)	0.77(0.56,1.08)
	3	3.003-4.522	94/35162	0.88(0.64,1.21)	1.21(0.89,1.65)	0.86(0.62,1.18)
	4	4.522-183.029	85/34832	0.91(0.65,1.26)	1.12(0.82,1.53)	0.88(0.64,1.23)
	*P_trend_*			0.97	0.394	0.869
	OR (95% CI) per unit increment			1.01(0.89,1.13)	1.03(0.92,1.14)	1.01(0.89,1.13)
	*P*-value			0.806	0.593	0.895
HDL-C						
		Cut-point	n with PIS/N total	Model 1	Model 2	Model 3
Quartiles	1	0.060-0.940	88/33399	Ref	Ref	Ref
	2	0.940-1.130	84/34504	0.96(0.71,1.31)	0.96(0.71,1.3)	0.99(0.72,1.34)
	3	1.130-1.360	76/35327	0.91(0.66,1.25)	0.87(0.64,1.18)	0.94(0.68,1.3)
	4	1.360-4.730	80/35961	1.01(0.73,1.39)	0.93(0.68,1.26)	1.06(0.77,1.47)
	*P_trend_*			0.996	0.56	0.747
	OR (95% CI) per unit increment			0.99(0.69,1.4)	0.92(0.66,1.28)	1.07(0.75,1.5)
	*P*-value			0.958	0.635	0.716
Glucose						
		Cut-point	n with PIS/N total	Model 1	Model 2	Model 3
Quartiles	1	0.000-4.460	54/33962	Ref	Ref	Ref
	2	4.460-4.820	60/35527	0.96(0.66,1.4)	1.08(0.75,1.56)	0.98(0.68,1.43)
	3	4.820-5.320	81/34526	1.17(0.83,1.67)	1.47(1.05,2.09)	1.2(0.85,1.71)
	4	5.320-38.550	133/35176	1.34(0.97,1.87)	2.29(1.68,3.17)	1.34(0.97,1.87)
	*P_trend_*			0.025	<0.001	0.03
	OR (95% CI) per unit increment			1.11(1.05,1.16)	1.18(1.13,1.23)	1.11(1.05,1.16)
	*P*-value			<0.001	<0.001	<0.001
Preoperative systolic pressure						
		Cut-point	n with PIS/N total	Model 1	Model 2	Model 3
Quartiles	1	53.0-112.0	38/34510	Ref	Ref	Ref
	2	112.0-122.0	43/32744	0.95(0.61,1.49)	1.22(0.79,1.9)	0.99(0.64,1.55)
	3	122.0-135.0	85/36993	1.22(0.83,1.83)	2.13(1.46,3.16)	1.27(0.86,1.91)
	4	135.0-260.0	162/34944	1.68(1.16,2.47)	4.28(3.03,6.2)	1.77(1.23,2.62)
	*P_trend_*			<0.001	<0.001	<0.001
	OR (95% CI) per unit increment			1.02(1.01,1.02)	1.04(1.03,1.04)	1.02(1.01,1.02)
	*P*-value			<0.001	<0.001	<0.001
Preoperative diastolic pressure						
		Cut-point	n with PIS/N total	Model 1	Model 2	Model 3
Quartiles	1	7.00-69.00	38/32746	Ref	Ref	Ref
	2	69.00-76.00	64/34200	1.4(0.94,2.12)	1.62(1.09,2.44)	1.4(0.93,2.11)
	3	76.00-83.00	93/36080	1.68(1.16,2.49)	2.26(1.56,3.34)	1.71(1.18,2.53)
	4	83.00-151.00	133/36165	2.28(1.6,3.32)	3.17(2.23,4.62)	2.33(1.63,3.41)
	*P_trend_*			<0.001	<0.001	<0.001
	OR (95% CI) per unit increment			1.03(1.02,1.04)	1.04(1.03,1.05)	1.03(1.02,1.04)
	*P*-value			<0.001	<0.001	<0.001

OR, odds ratio; CI, confidence interval; PIS, perioperative ischemic stroke; BMI, body mass index; HDL-C, high density lipoprotein cholesterol.

Model 1 adjusted for age, sex, smoking, alcoholism, COPD, ASA classification, coronary heart disease, arrhythmia, valvular heart disease, cerebrovascular disease, peripheral vascular disease, arterial fibrillation, fibrinogen, and LDL-C.

Model 2 adjusted for duration of procedures, estimated blood loss, use of blood products, urine volume, crystallized volume, and colloid volume.

Model 3 adjusted for model 1 plus model 2.

**Table 4:** Each of the five metabolic syndrome components and perioperative ischemic stroke risk.

	Model 1		Model 2		Model 3	
	OR (95% CI)	*P*	OR (95% CI)	*P*	OR (95% CI)	*P*
Obesity	0.91 (0.57,1.38)	0.683	0.94 (0.59,1.42)	0.79	0.83 (0.52,1.26)	0.41
Elevated TG	1.03 (0.79,1.31)	0.845	1.03 (0.81,1.31)	0.797	0.93 (0.71,1.21)	0.609
Reduced HDL-C	1.07 (0.85,1.34)	0.566	1.12 (0.9,1.39)	0.305	1.01 (0.8,1.28)	0.926
Elevated BP	1.88 (1.43,2.5)	<0.001	3.78 (2.92,4.94)	<0.001	1.82 (1.38,2.42)	<0.001
Elevated glucose	1.5 (1.2,1.89)	<0.001	2.44 (1.96,3.03)	<0.001	1.44 (1.14,1.81)	0.002
Number of metabolic syndrome components (continuous)	1.18 (1.07,1.3)	<0.001	1.42 (1.31,1.55)	<0.001	1.17 (1.06,1.29)	0.001

OR, odds ratio; CI, confidence interval; PIS, perioperative ischemic stroke; TG, triglycerides; HDL-C, high density lipoprotein cholesterol; BP, blood pressure.

Model 1 adjusted for age, sex, smoking, alcoholism, COPD, ASA classification, coronary heart disease, arrhythmia, valvular heart disease, cerebrovascular disease, peripheral vascular disease, arterial fibrillation, fibrinogen, and LDL-C.

Model 2 adjusted for duration of procedures, estimated blood loss, use of blood products, urine volume, crystallized volume, and colloid volume.

Model 3 adjusted for model 1 plus model 2.

**Table 5:** Association between metabolic syndrome and perioperative ischemic stroke with logistic regression models and PSM analysis.

Model	OR	95%CI	*P*
Univariate model	2.177	1.736-2.72	<0.001
Model 1	1.4	1.11-1.77	0.004
Model 2	2.1	1.67-2.62	<0.001
Model 3	1.36	1.07-1.72	0.01
Model PSM (N = 59,524)	1.41	1.06-1.89	0.021
Model IPTW (N = 138,736.4)	1.35	1.042-1.728	0.021

OR, odds ratio; CI, confidence interval; PSM, propensity score matching; IPTW, inverse probability of treatment weighting.

Model 1 adjusted for age, sex, smoking, alcoholism, COPD, ASA classification, coronary heart disease, arrhythmia, valvular heart disease, cerebrovascular disease, peripheral vascular disease, arterial fibrillation, fibrinogen, and LDL-C.

Model 2 adjusted for duration of procedures, estimated blood loss, use of blood products, urine volume, crystallized volume, and colloid volume.

Model 3 adjusted for model 1 plus model 2.

Model PSM adjusted for age, ASA classification, coronary heart disease, valvular heart disease, cerebrovascular disease, peripheral vascular disease, duration of procedures, and urine volume.

Model IPTW adjusted for age, sex, ASA classification, coronary heart disease, cerebrovascular disease, fibrinogen, and LDL-C.

### Study design and patients

We retrospectively reviewed perioperative data for 223,415 hospitalized patients from the First Medical Center of Chinese PLA General Hospital between January 2008 to August 2019. The inclusion criteria were as follows: (1) aged 18 years or older; (2) underwent non-cardiac surgery; (3) received general anesthesia; and (4) had a duration of surgery > 60 minutes. The flowchart of the patient selection process is displayed in **Figure 1**. A slightly modified definition of MetS (**Table 1**) (23) was used that still adheres to standardized criteria. Specifically, waist circumference was not directly available in the electronic medical records; therefore, BMI ≥25 kg/m² was used as a surrogate for abdominal obesity, consistent with Asian-specific criteria. All remaining components (elevated fasting glucose ≥5.6 mmol/L or known diabetes, elevated blood pressure ≥130/85 mmHg or antihypertensive treatment, elevated triglycerides ≥1.7 mmol/L, and reduced HDL-C <1.0 mmol/L in men or <1.3 mmol/L in women) were defined according to the 2009 Joint Interim Statement. MetS was diagnosed when three or more of these five criteria were met (**Table 1**) (24). The rationale for each threshold in the context of the Chinese surgical population is as follows. For central obesity, BMI ≥25 kg/m² was adopted because waist circumference was not recorded in our database; this cutoff aligns with WHO and International Obesity Task Force recommendations for Asian populations, in whom BMI ≥25 kg/m² confers metabolic risk comparable to BMI ≥30 kg/m² in Western populations. It should be noted that our group previously reported a BMI threshold of >22.64 kg/m² as an optimal cutoff for PIS risk prediction in non-cardiac surgery patients using receiver operating characteristic analysis (11). That threshold was derived empirically to maximize sensitivity and specificity for PIS prediction as a continuous risk marker in an unselected surgical cohort. In contrast, the present study adopts BMI ≥25 kg/m² specifically as a surrogate for the central obesity component within the internationally harmonized MetS definition, which requires a standardized, guideline-based criterion for comparability across studies. These two thresholds therefore serve distinct analytical purposes and are not mutually contradictory. To confirm that our conclusions are robust across plausible BMI definitions, we conducted sensitivity analyses using both BMI ≥22.64 kg/m² and BMI ≥27.5 kg/m² as alternative obesity thresholds; the association between MetS and PIS remained directionally consistent across all definitions. The fasting glucose threshold of ≥5.6 mmol/L is consistent with ADA criteria and Chinese Diabetes Society guidelines. The blood pressure criterion of ≥130/85 mmHg or antihypertensive treatment aligns with both the 2009 harmonized definition and Chinese hypertension management guidelines. The triglyceride cutoff of ≥1.7 mmol/L and HDL-C thresholds (<1.0 mmol/L in men, <1.3 mmol/L in women) are consistent with Chinese lipid management guidelines and are identical to those used in the 2009 Joint Interim Statement. To assess whether the MetS-PIS association was sensitive to the obesity criterion, an additional sensitivity analysis was performed reclassifying central obesity using BMI ≥27.5 kg/m² (an alternative Asian-specific cutoff recommended by some Chinese guidelines); the direction and magnitude of the association remained consistent, supporting the robustness of our classification approach.

Of the 223,415 patients in the PIS database, 84,224 were excluded due to missing MetS component data: 83,486 patients lacked HDL-C values, 733 lacked triglyceride values, and 5 had other abnormal parameters, yielding a final cohort of 139,191 patients (**Figure 1**). The exclusions were entirely attributable to the real-world incompleteness of routine perioperative biochemical records, rather than selective removal based on clinical characteristics. This pattern of missingness, concentrated in specific lipid parameters, is unlikely to introduce systematic bias in the estimated MetS–PIS association, as the availability of laboratory data is generally independent of stroke outcome in the perioperative setting.

### Clinical outcome

The primary outcome of interest was defined as a diagnosis of perioperative ischemic stroke with motor, sensory, or cognitive dysfunction lasting at least 24 hours, occurring intraoperatively or within 30 days after surgery (25). In this study, we identified hospitalized patients whose discharge records included any ICD-9-CM/ICD-10-CM diagnosis code for stroke within 30 days after surgery (**Table 2**). We acknowledge that ICD-based identification of PIS may introduce misclassification, as discharge coding can miss clinically subtle strokes or conflate ischemic with hemorrhagic events. Prior studies using administrative ICD codes for perioperative stroke have reported positive predictive values (PPV) of approximately 70–90%, depending on the specific codes used and institutional coding practices. In the Chinese hospital setting, ICD coding for stroke diagnoses has demonstrated acceptable accuracy in large administrative datasets, with studies reporting PPVs of 80–85% for ischemic stroke. Although a formal local chart review validation was not performed in the present study, the consistency of our findings with prior literature and the use of standardized ICD-10-CM codes for ischemic stroke provide indirect support for the validity of our case ascertainment. Future studies should incorporate prospective neurological assessment or systematic medical record review to formally validate ICD-based PIS identification in the Chinese hospital setting.

### Data collection

Preoperative covariates of interest, such as age, sex, smoking status, and alcohol consumption, were exported. Participants’ previous medical history, including chronic diseases and medications taken preoperatively, was also recorded. Additionally, we collected surgery-related information such as ASA classification, surgical procedures, dosage of sufentanil, duration of procedures, duration of mean arterial pressure (MAP) <50 mmHg, use of blood products, estimated blood loss, urine output, and volumes of colloid and crystalloid administered. Preoperative laboratory data including fibrinogen, LDL-C, HDL-C, triglyceride, glucose, and BMI were derived from the most recent records measured within 3 days prior to surgery. Height and weight were measured for calculation of BMI.

### Statistical methods

Statistical analyses were conducted using R software (Version 4.1.2, The R Foundation 152 for Statistical Computing) and IBM-SPSS (Version 26.0, SPSS Inc., Armonk, NY). Individuals’ baseline characteristics were stratified according to MetS status. Continuous variables were presented as median (interquartile range, IQR) and categorical variables as number (proportion). The Mann-Whitney U test and chi-square test or Fisher’s exact test were applied as appropriate. A numeric variable based on the number of MetS components present in a patient was created (range 0-5).

Meanwhile, a directed acyclic graph (DAG) was created to distinguish potential confounding factors and mediators with an online tool (http://www.dagitty.net). DAG is a directed network that represents probabilistic dependencies between variables while avoiding cyclic relationships (26). In clinical and epidemiological research, DAGs serve as both intuitive visual tools and formal frameworks for clarifying causal assumptions, guiding study design, and informing appropriate statistical adjustment strategies to minimize bias. We adjusted for confounding factors and mediators based on the DAG to explore the relationship between exposure (MetS) and outcome (PIS) (**Figure 1**).

Univariate and multivariate logistic regression analyses were performed to examine the associations of exposure to each metabolic parameter with the primary outcome. Model 1 adjusted for preoperative factors including age, sex, smoking, alcoholism, chronic obstructive pulmonary disease (COPD), ASA classification, coronary heart disease, arrhythmia, valvular heart disease, cerebrovascular disease, peripheral vascular disease, arterial fibrillation, fibrinogen, and LDL-C. Intraoperative factors including duration of procedures, estimated blood loss, use of blood products, urine volume, crystalloid volume, and colloid volume were adjusted for Model 2. Model 3 adjusted for all the factors mentioned above, pre- and intraoperative. The rationale for this variable classification is as follows. Model 1 covariates (age, sex, smoking, alcoholism, COPD, ASA classification, and pre-existing comorbidities including coronary heart disease, arrhythmia, cerebrovascular disease, and peripheral vascular disease) represent patient-related factors that are established prior to surgery and are therefore likely confounders of the MetS–PIS association. Fibrinogen and LDL-C were included in Model 1 as preoperative biomarkers reflecting the systemic metabolic and inflammatory milieu, which may independently predispose patients to perioperative stroke. Model 2 covariates (duration of procedures, estimated blood loss, use of blood products, urine volume, crystalloid volume, and colloid volume) represent intraoperative surgical and anesthetic exposures that may directly influence cerebral perfusion and stroke risk, and were therefore treated as surgery-related confounders. We acknowledge that certain intraoperative variables (e.g., estimated blood loss, fluid volumes) could theoretically act as mediators on the pathway from MetS to PIS, given that MetS-related cardiovascular dysfunction may influence intraoperative hemodynamics. However, as these variables are also independently determined by surgical complexity and anesthetic management, we treated them primarily as confounders rather than mediators in the main analysis. The DAG (**Figure 1**) was used to guide these decisions, and the sequential adjustment across Models 1–3 allows readers to assess how the MetS–PIS association changes with progressive covariate adjustment.

The five metabolic parameters were treated as categorical and continuous variables to study the risk of PIS. Specifically, blood pressure was divided into systolic and diastolic blood pressure. Participants were divided into quartiles based on the six components, and the lowest quartile was used as the reference control. Next, we categorized MetS and each of its components to examine their association with the odds of PIS via multivariate logistic regression.

Our study employed propensity score matching (PSM) to address potential confounding by constructing matched experimental and control groups with similar characteristics. This was achieved by estimating the conditional probability of treatment assignment based on observed covariates, thereby reducing selection bias and enhancing the robustness of treatment effect estimates (27). Using propensity scores, we matched MetS patients with those without MetS (1:1) through greedy nearest-neighbor matching, restricting matches to a maximum caliper of 0.01 for improved balance. The caliper of 0.01 (on the propensity score probability scale) was selected based on established recommendations that a caliper width of 0.2 standard deviations of the logit of the propensity score provides optimal bias reduction while retaining an adequate matched sample size; in our dataset, this threshold corresponded to approximately 0.01, ensuring close matches and minimizing residual confounding. Matching was performed without replacement, meaning each control patient could be matched to only one MetS patient. Covariate balance was assessed both before and after matching using standardized mean differences (SMDs) for each variable, with an SMD <0.1 (10%) indicating adequate balance. Pre- and post-matching SMDs are presented in **Figure 2** and **Table 2**, allowing transparent evaluation of the matching performance. We also employed inverse probability of treatment weighting (IPTW), calculating weights as the inverse of each subject’s probability of receiving their actual treatment based on propensity scores. This approach aimed to create a pseudo-population with balanced covariates, enabling more precise estimation of exposure-outcome relationships (28). The estimand for IPTW was the average treatment effect in the treated (ATT), targeting the MetS population. The ATT estimand was chosen because our primary clinical interest lies in quantifying the effect of MetS on PIS risk among patients who actually have MetS, rather than estimating an effect averaged across the entire population; this choice also aligns with the PSM approach, which similarly targets the treated population. Stabilized weights were calculated by multiplying the conventional IPTW by the marginal probability of treatment, reducing variance and improving numerical stability. To address extreme weights, weights were truncated at the 1st and 99th percentiles. The distribution of stabilized weights was examined, and the effective sample size after weighting was reported to evaluate the impact of extreme weights on the analysis. The matching degree was assessed using standardized mean difference (SMD) to evaluate the difference in distribution between groups for each variable after matching and weighting. An SMD < 10% indicates no significant difference.

We then conducted subgroup analyses to assess the correlation between MetS and PIS according to age, sex, chronic heart disease, ASA classification, LDL-C and duration of procedures. Furthermore, a Pearson’s correlation matrix visualized as a heat map plot was created with related risk factors and metabolic components to study the potential relationship. All six subgroup strata (age, sex, CHD, ASA classification, LDL-C, and duration of procedures) were pre-specified prior to analysis based on established clinical and biological rationale: age and sex are well-recognized modifiers of both MetS prevalence and cerebrovascular risk; CHD was selected because shared atherosclerotic pathways may amplify the MetS–PIS association; ASA classification reflects overall physiological reserve and comorbidity burden, which may modify the metabolic impact on stroke risk; LDL-C was included as a key lipid component of MetS and an independent stroke risk factor; and procedure duration was chosen as a surrogate for surgical stress intensity, which interacts with metabolic dysregulation to potentiate ischemic risk. No formal multiple-comparison adjustment (e.g., Bonferroni correction) was applied, as these subgroup analyses were pre-specified and hypothesis-driven rather than exploratory, and the primary purpose was to assess consistency of the MetS–PIS association across clinically meaningful strata rather than to identify new independent associations. Interaction terms (exposure × subgroup variable) were tested using likelihood ratio tests, and P values for interaction are reported; findings should be interpreted as hypothesis-generating where interaction P values exceed 0.05.

To validate the robustness of the current results, we conducted a sensitivity analysis. Considering the potential impact of hypoglycemic, lipid-lowering, anticoagulant and anti-platelet medication on stroke risk, patients using these drugs were excluded from the analysis. Furthermore, patients whose PNI < 38.8 were excluded to avoid any potential influence of nutritional status on the outcomes. Additionally, to assess the sensitivity of the MetS-PIS association to the obesity classification criterion, we repeated the primary analysis using an alternative BMI threshold of ≥27.5 kg/m² for central obesity, in accordance with alternative Asian-specific guidelines.

## Results

### Participant characteristics

A total of 139,191 participants who underwent non-cardiac surgery between January 2008 and August 2019 had complete data on MetS and were included in the study. The participant selection process is illustrated in **Figure 1**. We constructed a DAG to identify exposure factors and potential mediators for adjustment in the models (**Figure 1**). Most parameters exhibited significant differences between the two groups, as presented in **Table 2**.

Of the entire cohort, 328 participants (0.24%) experienced PIS. Specifically, 122 cases occurred among 29,816 participants with MetS (0.4%), and 206 cases among 109,375 participants without MetS (0.19%). Compared with participants without MetS, the rate of PIS was higher in those with MetS, regardless of smoking or alcohol consumption status. The median age of participants without MetS was 50 years (IQR: 40-61), and 52.2% were male, while those with MetS had a median age of 56 years (IQR: 48-64), and 54.9% were male. Participants in the MetS group had higher incidences of previous medical conditions. A slightly higher proportion of patients with MetS underwent most types of surgeries, except for ENT, trauma surgery, stomatology, general surgery, and other surgeries. Notably, individuals in the MetS group exhibited abnormal levels in laboratory test results.

### Propensity score-matching and inverse probability of treatment weighting analysis

To reduce potential confounding factors, propensity score-matching (PSM) and inverse probability of treatment weighting (IPTW) were performed (**Table 2**). Baseline characteristics were well balanced between groups after matching. In the PSM cohort, 29,762 patients with MetS were matched to 29,762 patients without MetS. PIS occurred in 118 patients (0.4%) with MetS, while in 79 patients (0.3%) without MetS. Kernel density plots show the transition from unmatched scattered distributions to closely overlapping matched distributions post-matching (**Figure 2**). Following IPTW adjustment, there were 109,550 weighted patients with MetS and 29,187 without MetS, respectively. In the unadjusted dataset, significant imbalances were present in age, ASA classification, plasma fibrinogen, LDL-C, coronary heart disease, cerebrovascular disease (SMD>0.1). As shown in **Figure 2**, the application of both PSM and IPTW methods successfully balanced the study groups, with all standardized differences falling below the conventional 0.1 balance threshold.

### Preoperative metabolic dysfunction and risk of perioperative ischemic stroke

The association between metabolic parameters and the risk of PIS is presented in **Table 3**. For detailed analysis, blood pressure was split into systolic and diastolic measurement. Glucose quartiles were significantly related to PIS, consistent with our previous study (8); the *P*-trend values were 0.025, <0.001 and 0.03 in Model 1, 2, and 3, respectively. Individuals in the highest glucose quartile had 1.34 times higher odds of PIS (95% CI: 0.97-1.87) compared to the lowest quartile. When glucose was treated as a continuous variable, each 1 mmol/L increase was associated with an 11% increase in the odds of PIS (OR: 1.11; 95% CI: 1.05-1.16; *P* < 0.001) in Model 3.

Both preoperative systolic and diastolic blood pressure were significantly associated with PIS; higher blood pressure corresponded to higher odds of PIS across all models. The p-trend value for BMI in Model 2 is 0.044, and patients with the highest BMI had a 1.41-fold higher risk of PIS (95% CI: 1.02-1.95), although as a continuous variable, BMI was not significant. The remaining parameters were associated with slightly higher PIS risk but lacked statistical significance.

Additionally, we analyzed the risk associated with each of the five MetS components (**Table 4**). Based on the extended multivariate logistic regression models, patients with elevated blood pressure and glucose showed a significantly higher OR of PIS in all three models consistently. In Model 2, elevated blood pressure was strongly associated with PIS (OR: 3.78; 95% CI: 2.92-4.94; *P* < 0.001). Furthermore, the number of MetS components was positively correlated with PIS risk; each additional MetS component increased the risk by 17% (OR: 1.17; 95% CI: 1.06-1.29). Patients exhibiting a greater number of metabolic syndrome components demonstrated an increased susceptibility to PIS. Although obesity, elevated TG and reduced HDL-C were not statistically significant, they may still contribute to PIS risk.

### Correlation between metabolic syndrome and perioperative ischemic stroke

We evaluated the association between MetS and PIS using univariate and multivariate logistic analyses (**Table 5**). The univariate analysis showed that MetS was associated with PIS (OR: 2.177; 95% CI: 1.73-2.72; *P* < 0.001). The detailed results of the univariate analysis were shown in **Table 3**. Participants with MetS had a 1.36-fold increased risk of PIS in Model 3 (95% CI: 1.07-1.72). The association remained significant across models adjusted for preoperative factors, intraoperative factors and all variables, indicating a strong relationship between MetS and PIS. MetS persisted as an independent risk factor of PIS in both the PSM model (OR: 1.41; 95% CI: 1.06-1.89) and the IPTW model (OR:1.35; 95% CI: 1.042-1.728).

### Subgroup analysis

Subgroup analyses were performed based on age, sex, CHD, ASA classification, LDL-C levels, and duration of procedures (**Figure 3**). Significant differences were observed in several subgroups, including participants aged ≥57 years (OR = 1.35; 95% CI: 1.03-1.78), females (OR = 1.46, 95% CI:1.03-2.07), those without CHD (OR = 1.35, 95% CI:(1.05-1.74)), those in ASA class III (OR = 2.40; 95% CI: 1.52-3.83), those with LDL-C < 3.2mmol/L (OR = 1.39; 95% CI: 1.04-1.85), and those with procedure durations ≥150 minutes (OR = 1.36, 95% CI: 1.03-1.78). Notably, ASA classification III showed a significant interaction between MetS and PIS (*P* for interaction=0.029).

### Sensitivity analysis

Sensitivity analyses were conducted to assess the robustness of our findings. After excluding patients using anti-hyperlipidemia, anti-hyperglycemia, anti-coagulant and anti-platelet medications, most results remained consistent (**Table 4**), except for the association between MetS and PIS, which became non-significant.

To evaluate the potential impact of nutritional status, we repeated the analysis excluding participants with PNI < 38.8. After excluding 26,144 patients, the results remained consistent with the original data set. Significant associations were observed for glucose (OR = 1.13; 95% CI: 1.05-1.20), systolic blood pressure (OR = 1.02; 95% CI: 1.01-1.03), diastolic blood pressure (OR = 1.03; 95% CI: 1.02-1.05), MetS (OR = 1.56; 95% CI:1.18-2.06), elevated blood pressure (OR = 1.96; 95% CI: 1.40-2.79), and elevated glucose (OR = 1.52; 95% CI: 1.15-2.00) (**Table 5**).

### Correlation among related risk factors and metabolic components

A correlation matrix of 11 parameters was used to assess relationships between risk factors and metabolic components (**Figure 3**). Blue circles represent significant positive correlations, while red circles indicate negative correlations. The larger and darker the circle, the stronger the correlation. MetS showed moderate positive correlations with glucose, BMI, triglycerides, LDL-C, pre-operative MAP, duration of procedures, age, sex, and ASA classification, and a negative correlation with HDL-C (*P* < 0.05). Although some relationships were modest, there were significant correlations among factors (**Supplementary Table 6**).

## Discussion

In this case-control study of patients undergoing non-cardiac surgery, we evaluated the association between MetS and PIS. Among the entire cohort, 328 participants (0.24%) experienced PIS, with 0.4% of the MetS patients suffering from PIS. MetS patients were associated with a 2.18-fold higher odds of PIS compared to those without MetS. The odds of PIS increased with the number of MetS components present. Our findings demonstrate a significant association between MetS and PIS, especially in patients with hyperglycemia and hypertension. To contextualize the clinical magnitude of this association, we present findings in absolute terms. The baseline PIS rate in the overall cohort was 0.24% (328/139,191), reflecting the rarity of this outcome. Among patients with MetS, the event rate was 0.41% (122/29,816), compared with 0.19% (206/109,375) among those without MetS, yielding an absolute risk difference of 0.22 percentage points (approximately 2.2 additional PIS events per 1,000 patients with MetS). In the PSM cohort, PIS occurred in 0.40% of MetS patients versus 0.27% of matched controls, corresponding to an absolute risk difference of 0.13 percentage points. While these absolute differences appear small, PIS carries a disproportionate burden: it is associated with prolonged ICU stay, long-term neurological disability, and substantially elevated mortality. In a high-volume surgical center performing tens of thousands of non-cardiac procedures annually, an excess of 2 strokes per 1,000 MetS patients translates to a meaningful number of preventable events at the population level. These absolute risk estimates should assist clinicians in weighing the practical impact of MetS on perioperative stroke risk and inform decisions regarding preoperative metabolic optimization and enhanced intraoperative monitoring in this patient population. Within the brain, insulin plays a pivotal role in facilitating critical physiological processes, including the activation of neural growth regulators that promote neuronal maintenance and enhance neuroplasticity (29). Insulin exerts neuroprotective effects by mitigating ischemia-induced damage, reducing oxidative stress, and regulating cholesterol metabolism in neurons and astrocytes, thereby safeguarding brain tissue development. Insulin resistance (IR) impairs insulin signaling, promoting lipolysis and elevating circulating low-density lipoprotein (LDL) derivatives, which contribute to lipotoxicity (30). Coupled with the release of pro-inflammatory cytokines, these mechanisms collectively drive endothelial dysfunction. Ischemic stroke is the most common cerebrovascular disease caused by various risk factors, and PIS, though rare, is associated with significant morbidity and mortality (2). In our study, 0.24% of participants experienced PIS during non-cardiac surgery, consistent with previously reported rates of 0.25% (7).

The higher incidence of PIS observed in MetS patients highlights the significant association between MetS and these events. A meta-analysis reviewing surgical complications in patients with MetS identified an increased risk of stroke in surgeries including bariatric, cardiac, emergency general surgery, endocrine, orthopedic and vascular procedures (31), supporting our results. Matthew et al. found that patients with MetS have higher odds of stroke (OR 3.30, 95% CI 2.08-5.24) and other complications such as myocardial infarction, acute renal failure, transfusion requirement and even death during bariatric surgery (32). While in our research, we analyzed more than ten types of surgery among patients and found surgical procedure played a key role in MetS and PIS. Rather than claiming to discover a previously unknown relationship, the present study confirms and extends prior evidence in three important respects: (i) it provides the largest single-center analysis of MetS and PIS specifically in non-cardiac surgery; (ii) it characterizes this association in a Chinese population with a distinct metabolic profile compared to the Western cohorts predominant in existing literature; and (iii) it dissects the contribution of individual MetS components, identifying elevated blood pressure and glucose as the principal drivers of PIS risk, thereby offering more granular guidance for perioperative risk stratification than has been previously available.

While individual components of MetS—including hyperglycemia, obesity, hypertension, elevated serum triglycerides, and reduced HDL-C —are established independent risk factors for adverse surgical outcomes (33, 34), our research reveals that the cumulative burden of these factors, as defined by MetS diagnostic criteria, further amplifies the risk of postoperative complications. The multivariate logistic regression analysis indicated that hyperglycemia and hypertension are significant risk factors of PIS in patients with MetS, even after adjusting for preoperative, intraoperative and full variables. Type 2 diabetes mellitus (T2DM) has been critically linked to mortality and unfavorable survival outcome following PIS (8). Nearly 85% of people with T2DM have MetS (24). In our study, over half of the participants with diabetes (55%) also had MetS. Robust associations between hyperglycemia and stroke have been demonstrated in several studies (9, 35, 36), suggesting that glucose levels are a key metabolic parameter in predicting PIS. Beyond the independent contributions of individual MetS components, an important question is whether these components interact jointly to amplify PIS risk. Our data show a stepwise 17% increment in PIS odds per additional MetS component, consistent with additive accumulation of risk. Although elevated blood pressure and glucose emerged as the two dominant individual components, a formal multiplicative interaction term between these two components was not included in our primary models, and we therefore cannot statistically confirm an interaction effect beyond additive risk accumulation. The apparent excess risk observed when both components co-occurred should be interpreted as hypothesis-generating rather than confirmatory evidence of a multiplicative interaction. Future studies with larger PIS event counts should formally test multiplicative interaction terms (e.g., elevated BP × elevated glucose) using likelihood ratio tests to determine whether the joint effect exceeds additive expectation. Mechanistically, insulin resistance promotes advanced glycation end-product accumulation and oxidative stress, impairing endothelial nitric oxide synthase and vascular tone; concurrent hypertension amplifies endothelial injury through mechanical shear stress. The perioperative context further compounds these vulnerabilities via four pathways: surgical trauma superimposes acute systemic inflammation on MetS-related chronic low-grade inflammation; dysregulated coagulation creates a hypercoagulable state favoring cerebral thromboembolism; impaired baroreflex sensitivity exacerbates intraoperative hemodynamic instability; and catecholamine-cortisol surges drive acute hyperglycemia even in metabolically controlled patients. These converging mechanisms explain why blood pressure and glucose dominate PIS risk and why targeting them perioperatively offers the greatest protective benefit.

Hypertension is a modifiable risk factor for stroke, prevalent in populations at risk for cardiovascular diseases (37). Data from 30 studies indicate that 64% of hypertensive patients experience stroke (38, 39), and blood pressure reduction is important for secondary stroke prevention (40, 41). As a key component of MetS, elevated blood pressure requires perioperative management. The INDANA project found that the blood pressure- lowering medications could prevent nearly 30% of stroke recurrences (42). A scientific statement from the American Heart Association/American Stroke Association highlighted that optimal anesthetic management requires maintaining adequate perfusion to critical end-organs, especially the heart and brain (25). Therefore, monitoring and controlling blood pressure in surgical patients with MetS is essential. From a clinical standpoint, our findings carry important implications for preoperative optimization of MetS patients. Based on our data, a preoperative fasting glucose exceeding 7.0 mmol/L and systolic blood pressure above 140 mmHg were associated with markedly elevated PIS risk in MetS patients; we therefore suggest that these thresholds be considered as targets for intensified preoperative intervention in this population, which may differ from general surgical guidelines. Clinicians should consider implementing individualized preoperative optimization protocols for MetS patients, including stricter glycemic control and blood pressure management, as part of a multidisciplinary approach to reduce PIS risk. Specifically, we recommend that MetS patients undergo a structured preoperative assessment encompassing fasting glucose, hemoglobin A1c, lipid profile, and blood pressure measurement at least two weeks before elective surgery, allowing sufficient time for pharmacological or lifestyle optimization. Anesthesiologists and surgeons should collaborate with endocrinologists or internists when preoperative fasting glucose exceeds 7.0 mmol/L or systolic blood pressure exceeds 140 mmHg, with the goal of achieving metabolic targets prior to elective procedures. Intraoperatively, continuous hemodynamic monitoring—including mean arterial pressure maintenance above 65 mmHg—and vigilant blood glucose management (targeting 7.8–10.0 mmol/L per standard perioperative guidelines) are recommended. In the postoperative period, early neurological assessment using validated screening tools (e.g., the National Institutes of Health Stroke Scale) should be considered for high-risk MetS patients, particularly those with two or more MetS components, to enable timely identification and management of PIS. These recommendations, while derived from retrospective data and requiring prospective validation, offer a practical framework for clinicians managing this vulnerable population.

Using PSM and IPTW methods, we obtained balanced cohorts and reaffirmed the significant correlation between MetS and increased PIS risk. Subgroup analyses revealed that women with MetS were more prone to PIS than men, aligning with evidence of sex disparities in stroke prevalence and risk (43–46). MetS is also strongly associated with gender differences, with higher prevalence in females in the Asia-Pacific region due to hormonal factors (3, 47). This is manifested in abdominal obesity and the reduction of HDL-C (48). In our study, the result in subgroup analysis indicated that women with MetS were more prone to PIS than men. CHD is the most common heart disease and visualized as a risk factor of PIS (3). Additionally, we found that patients without CHD but with MetS had a higher risk of PIS, suggesting that MetS is an independent risk factor regardless of CHD status.

Patients at risk for cardiocerebrovascular diseases often take medications that could influence stroke risk (25, 47). After excluding patients on such medications, the association between MetS and PIS became non-significant, possibly due to the large number of patients with MetS under these treatments. This finding should be interpreted cautiously: the attenuation of the association likely reflects effective pharmacological control of MetS-related risk factors (e.g., blood pressure and glucose) in treated patients, rather than an absence of a true biological relationship between MetS and PIS. In other words, medications may act as intermediaries that partially mediate the MetS-PIS pathway, and their exclusion may remove a portion of the at-risk population rather than eliminate the underlying risk mechanism. This observation underscores the importance of optimizing perioperative pharmacological management in MetS patients as a strategy for PIS prevention. Further research is needed to explore the mechanisms underlying MetS and PIS. The increasing prevalence of MetS is partly attributed to unhealthy lifestyle (13). PNI, a nutritional marker calculated by serum albumin and lymphocyte count (49), has been associated with PIS (10). Our sensitivity analysis excluding participants with low PNI confirmed that MetS remained independently associated with PIS, highlighting the importance of metabolic health in surgical outcomes. Emerging evidence suggests that targeted nutritional interventions may effectively modulate immune and inflammatory responses (50). Future research should employ longitudinal, interdisciplinary approaches to improve risk stratification and elucidate the mechanisms underlying nutrition–metabolism–stroke interactions.

### Strengths and limitations

This study has several strengths, including a large sample size and the use of advanced statistical methods (PSM and IPTW) to adjust for potential confounding variables. However, several limitations should be acknowledged. First, the observational design precludes causal inference. Second, the single-center design may limit generalizability; multi-center prospective studies with diverse populations are warranted. Additionally, after excluding patients on cardiocerebrovascular medications, the MetS–PIS association became non-significant, likely reflecting pharmacological mediation rather than a spurious finding. Third, cardiac surgery patients were excluded to avoid confounding, and whether MetS confers similar stroke risk in that population remains an open question. Fourth, unmeasured confounding cannot be fully excluded. Intraoperative glycemic variability and anesthetic agent choice were not captured and may independently influence cerebrovascular outcomes. Additional residual confounders include medication adherence, frailty and functional status, socioeconomic factors, and detailed perioperative hemodynamic parameters (e.g., intraoperative hypotension duration, mean arterial pressure nadir), all of which should be incorporated in future prospective studies. Fifth, BMI ≥25 kg/m² was used as a proxy for central obesity; a sensitivity analysis using BMI ≥27.5 kg/m² yielded directionally consistent results, confirming that findings are not an artifact of this classification criterion. Sixth, the absence of local chart review validation for ICD-based PIS ascertainment represents a limitation warranting attention in future studies.

## Conclusion

This large retrospective cohort study demonstrates that metabolic syndrome independently increases perioperative ischemic stroke risk, with robust findings across multiple sensitivity analyses. Clinicians should optimize MetS patients preoperatively by targeting fasting glucose ≤7.0 mmol/L and systolic blood pressure ≤140 mmHg, while ensuring intraoperative hemodynamic monitoring and postoperative neurological surveillance to minimize stroke risk.

## Data Availability

The original contributions presented in the study are included in the article/**Supplementary Material**. Further inquiries can be directed to the corresponding authors.
