# Supplementary material for: Metabolic syndrome and the risk of perioperative ischemic stroke in non-cardiac surgery: a case-control retrospective study
Source: Front Endocrinol (Lausanne). 2026 Mar 11;17:1785277. doi: 10.3389/fendo.2026.1785277 (PMC13012999; doi:10.3389/fendo.2026.1785277)
Supplement: Supplementary file 1 [file DataSheet1.docx]

**Supplementary materials for review**

Figure S1. Directed acyclic graph of the link between metabolic syndrome and perioperative ischemic stroke.

Figure S2. Distribution of propensity scores in patients with and without MetS.

Figure S3. Correlation analysis among key perioperative factors and metabolic syndrome components.

Table S1. STROBE Statement—Checklist of Items that Should Be Included in Reports of Cohort Studies.

Table S2. ICD-9/10 Diagnosis Codes for Ischemic Stroke

Table S3. Univariate logistic regression analysis of variables for perioperative ischemic stroke.

Table S4. Association between metabolic syndrome and perioperative ischemic stroke in sensitivity analyses.

Table S5. Association between metabolic syndrome and perioperative ischemic stroke in sensitivity analyses.

Table S6. Correlation analysis among key perioperative factors and metabolic syndrome components.

**Figure S1. Directed acyclic graph of the link between metabolic syndrome and perioperative ischemic stroke.**

**
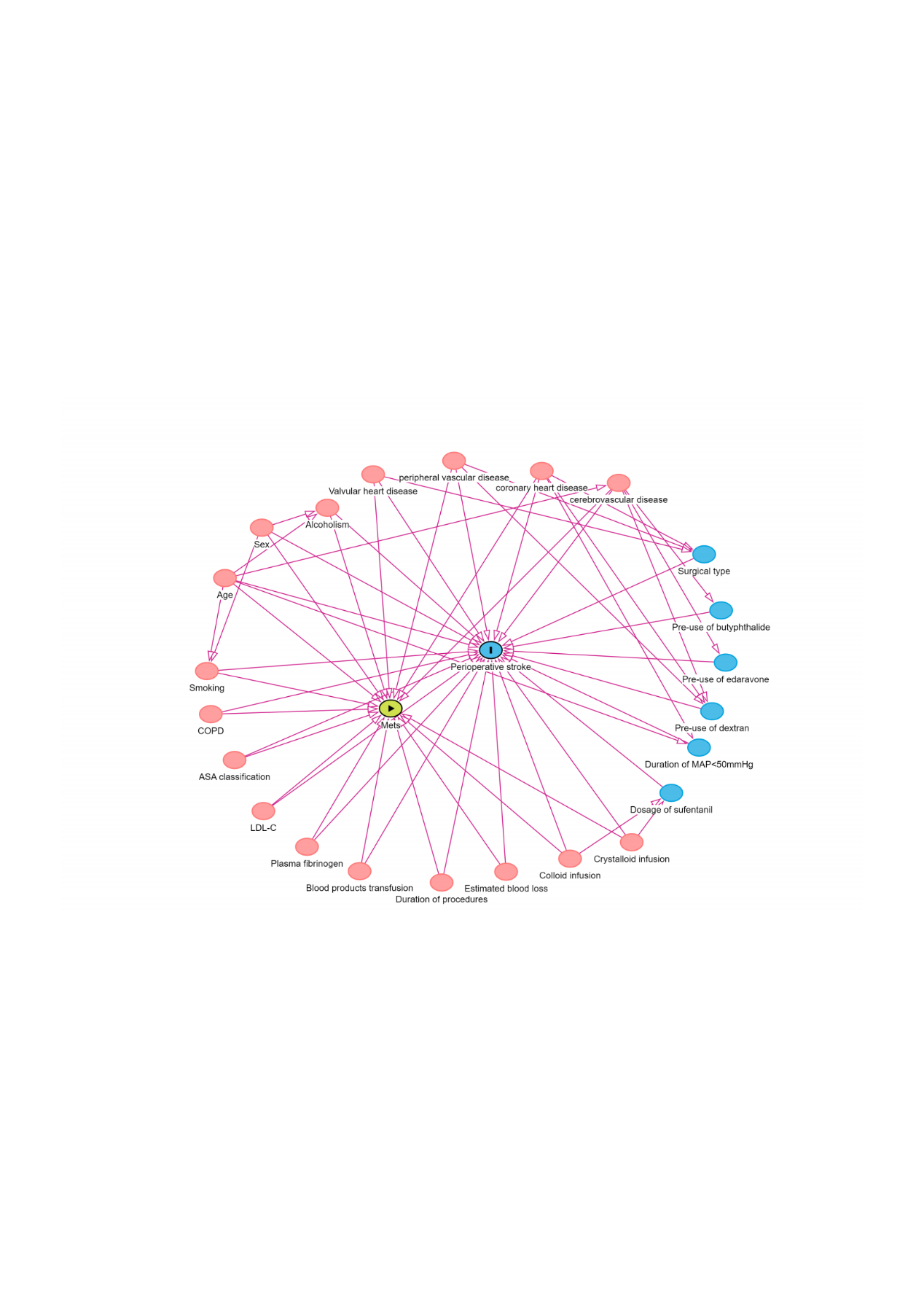
**

: mediator factous.

: confounding； factous;

: outcome;

: exposure;


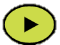

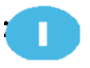

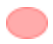

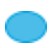


**Figure S2. Distribution of propensity scores in patients with and without MetS.**


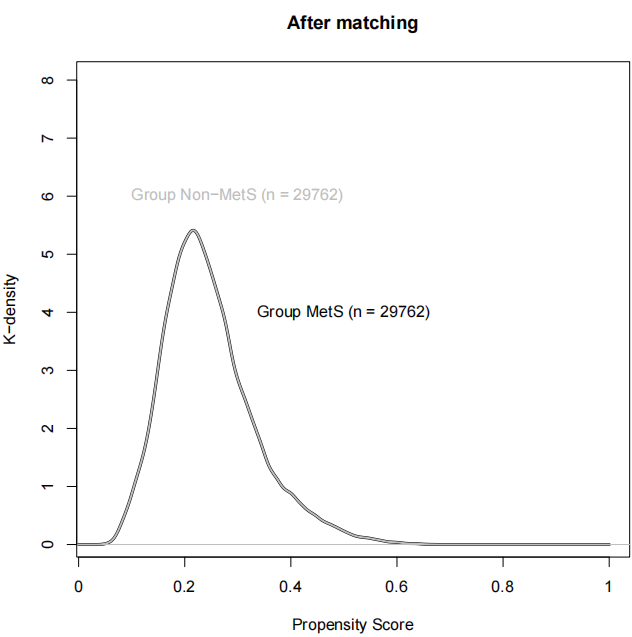

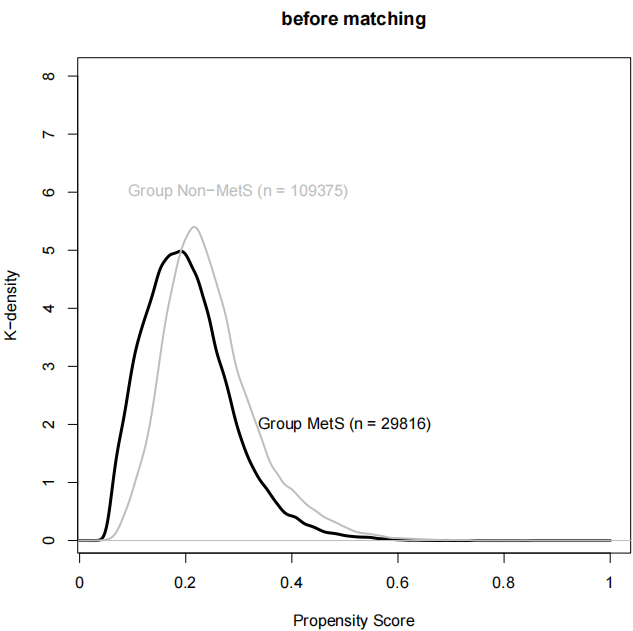


**Figure S3. Correlation analysis among key perioperative factors and metabolic syndrome components.**

**
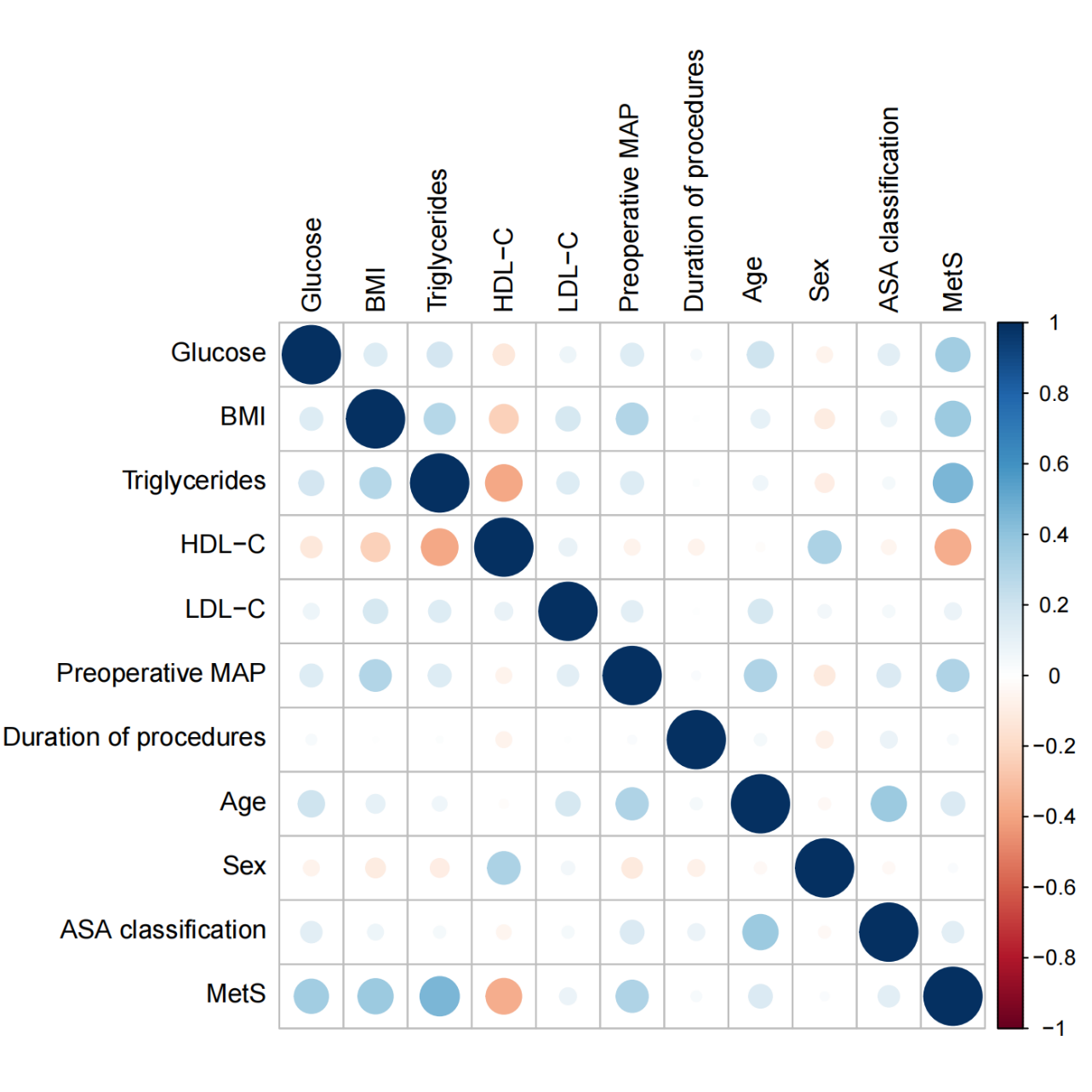
**A correlation matrix of 11 parameters was used to assess relationships between risk factors and metabolic components. Blue circles represent positive correlations, while red circles indicate negative correlations. The larger and darker the circle, the stronger the correlation.

**Table S1. STROBE Statement—Checklist of Items that Should Be Included in Reports of Cohort Studies**

|  | **Item No** | **Recommendation** | **Page No** |
| --- | --- | --- | --- |
| **Title and abstract** | 1 | (*a*) Indicate the study’s design with a commonly used term in the title or the abstract | 1 |
|  |  | (*b*) Provide in the abstract an informative and balanced summary of what was done and what was found | 2 |
| **Introduction** | | |  |
| Background/rationale | 2 | Explain the scientific background and rationale for the investigation being reported | 2 |
| Objectives | 3 | State specific objectives, including any prespecified hypotheses | 2 |
| **Methods** | | |  |
| Study design | 4 | Present key elements of study design early in the paper | 3 |
| Setting | 5 | Describe the setting, locations, and relevant dates, including periods of recruitment, exposure, follow-up, and data collection | 3 |
| Participants | 6 | (*a*) Give the eligibility criteria, and the sources and methods of selection of participants. Describe methods of follow-up | 3 |
|  |  | (*b*) For matched studies, give matching criteria and number of exposed and unexposed | 3 |
| Variables | 7 | Clearly define all outcomes, exposures, predictors, potential confounders, and effect modifiers. Give diagnostic criteria, if applicable | 3 |
| Data sources/ measurement | 8* | For each variable of interest, give sources of data and details of methods of assessment (measurement). Describe comparability of assessment methods if there is more than one group | 3,4 |
| Bias | 9 | Describe any efforts to address potential sources of bias | 4 |
| Study size | 10 | Explain how the study size was arrived at | 3,4 |
| Quantitative variables | 11 | Explain how quantitative variables were handled in the analyses. If applicable, describe which groupings were chosen and why | 3,4 |
| Statistical methods | 12 | (*a*) Describe all statistical methods, including those used to control for confounding | 3,4 |
|  |  | (*b*) Describe any methods used to examine subgroups and interactions | 3,4 |
|  |  | (*c*) Explain how missing data were addressed | Not applicable |
|  |  | (*d*) If applicable, explain how loss to follow-up was addressed | Not applicable |
|  |  | (*e*) Describe any sensitivity analyses | 4 |
| **Results** | | |  |
| Participants | 13* | (a) Report numbers of individuals at each stage of study—eg numbers potentially eligible, examined for eligibility, confirmed eligible, included in the study, completing follow-up, and analysed | 4,5 |
|  |  | (b) Give reasons for non-participation at each stage | Not applicable |
|  |  | (c) Consider use of a flow diagram | Figure 1 |
| Descriptive data | 14* | (a) Give characteristics of study participants (eg demographic, clinical, social) and information on exposures and potential confounders | Table 1 |
|  |  | (b) Indicate number of participants with missing data for each variable of interest | Not applicable |
|  |  | (c) Summarise follow-up time (eg, average and total amount) | Not applicable |
| Outcome data | 15* | Report numbers of outcome events or summary measures over time | 5 |
| Main results | 16 | (*a*) Give unadjusted estimates and, if applicable, confounder-adjusted estimates and their precision (eg, 95% confidence interval). Make clear which confounders were adjusted for and why they were included | 5,6  Table 2, Supplementary Table S3, Supplementary Table S4 |
|  |  | (*b*) Report category boundaries when continuous variables were categorized | 5 |
|  |  | (*c*) If relevant, consider translating estimates of relative risk into absolute risk for a meaningful time period | Not applicable |
| Other analyses | 17 | Report other analyses done—eg analyses of subgroups and interactions, and sensitivity analyses | 6 |
| **Discussion** | | |  |
| Key results | 18 | Summarise key results with reference to study objectives | 7,8 |
| Limitations | 19 | Discuss limitations of the study, taking into account sources of potential bias or imprecision. Discuss both direction and magnitude of any potential bias | 8 |
| Interpretation | 20 | Give a cautious overall interpretation of results considering objectives, limitations, multiplicity of analyses, results from similar studies, and other relevant evidence | 7,8 |
| Generalisability | 21 | Discuss the generalisability (external validity) of the study results | Not applicable |
| Other information | | |  |
| Funding | 22 | Give the source of funding and the role of the funders for the present study and, if applicable, for the original study on which the present article is based | 8 |

**Table S2. ICD-9/10 Diagnosis Codes for Ischemic Stroke**

| Ischemic stroke | ICD-9/ICD-10 | 433.X1/I63.X | Occlusion and stenosis of pre-cerebral arteries with cerebral infarction |
| --- | --- | --- | --- |
|  | ICD-9 | 434.X1 | Occlusion of cerebral arteries with cerebral infarction |
|  | ICD-9/ICD-10 | 437.1/I67.81,  I67.89 | Other generalized ischemic cerebrovascular disease |
|  | ICD-9/ICD-10 | 437.9/I67.9 | Unspecified cerebrovascular disease |

**Table S3. Univariate logistic regression analysis of variables for perioperative ischemic stroke.**

| **Characteristic** | **Estimate** | ***P* value** | **OR (95%CI)** |
| --- | --- | --- | --- |
| Demographics | | | |
| Age | 0.059 | <0.001 | 1.061 |
| Male | -0.061 | 0.58 | 0.94 |
| Smoking | | | |
| Ever | 0.155 | 0.505 | 1.167 |
| Now | -0.128 | 0.513 | 0.88 |
| Alcoholism | | | |
| Ever | 0.016 | 0.951 | 1.017 |
| Now | -0.425 | 0.032 | 0.654 |
| Previous medical history | | | |
| Coronary heart disease | 1.363 | <0.001 | 3.907 |
| Arterial fibrillation | 1.219 | 0.016 | 3.382 |
| Arrhythmia | 0.224 | 0.109 | 1.25 |
| Cerebrovascular disease | 2.974 | <0.001 | 19.562 |
| TIA | 2.782 | <0.001 | 16.153 |
| Ischemic stroke | 2.868 | <0.001 | 17.594 |
| Valvular heart disease | 1.692 | <0.001 | 5.429 |
| Peripheral vascular disease | 1.857 | <0.001 | 6.405 |
| COPD | -0.261 | 0.713 | 0.77 |
| Hypertension | 1.339 | <0.001 | 3.815 |
| Lipid-lowering medication | 1.68 | <0.001 | 5.365 |
| Anticoagulant medication | 1.14 | <0.001 | 3.126 |
| Hypoglycemic medication | 1.082 | <0.001 | 2.949 |
| ACEI medication | 0.822 | <0.001 | 2.275 |
| ARB medication | 0.871 | <0.001 | 2.39 |
| β-blockers medication | 1.295 | <0.001 | 3.65 |
| Aspirin medication | 2.658 | <0.001 | 14.271 |
| Butylphthalide medication | 2.634 | <0.001 | 13.933 |
| Edaravone medication | 1.998 | <0.001 | 7.374 |
| Dextran medication | 2.155 | <0.001 | 8.632 |
| Surgical related factors | | | |
| ASA classification |  |  |  |
| Ⅱ | 1.076 | <0.001 | 2.932 |
| Ⅲ | 2.359 | <0.001 | 10.577 |
| Surgical procedures | | | |
| Trauma surgery | -13.04 | 0 | 0.961 |
| Obstetrics | -0.006 | 0.989 | 0.994 |
| Intra-abdominal surgery | 0.139 | 0.568 | 1.149 |
| Joint arthroplasty | 0.501 | 0.12 | 1.65 |
| Spine | 0.973 | <0.001 | 2.647 |
| Stomatology | 0.223 | 0.48 | 1.25 |
| Urologic surgery | -0.215 | 0.466 | 0.806 |
| General surgery | -2.146 | 0.003 | 0.117 |
| Other (plastic surgery, etc) | -0.486 | 0.425 | 0.615 |
| Neurosurgery | 1.521 | <0.001 | 4.577 |
| Thoracic | 0.095 | 0.778 | 1.1 |
| Vascular | 1.405 | <0.001 | 4.075 |
| Dosage of sufentanil | 0.008 | <0.001 | 1.008 |
| Duration of procedures | 0.004 | <0.001 | 1.004 |
| Duration of MAP<50mmHg | 0.012 | <0.001 | 1.012 |
| Use of blood products | 0.811 | <0.001 | 2.25 |
| Estimated blood loss | 0 | <0.001 | 1 |
| Urine volume | 0 | <0.001 | 1 |
| Colloid volume | 0.001 | <0.001 | 1.001 |
| Crystalloid volume | 0 | <0.001 | 1 |

**Table S4. Association between metabolic syndrome and perioperative ischemic stroke in sensitivity analyses.**

|  | **Perioperative ischemic stroke** |  |
| --- | --- | --- |
|  | **OR (95% CI) per unit incrementor for presence of categorical exposure** | ***P* value** |
| BMI | 0.99(0.95,1.03) | 0.621 |
| Triglycerides | 1.02(0.88,1.17) | 0.745 |
| HDL-C | 1.02(0.68,1.17) | 0.925 |
| Glucose | 1.09(1,1.17) | 0.034 |
| SBP | 1.02(1.01,1.02) | <0.001 |
| DBP | 1.04(1.02,1.05) | <0.001 |
| MetS | 1.28(0.95,1.7) | 0.094 |
| Obesity | 0.55(0.26,1.01) | 0.08 |
| Elevated TG | 0.94(0.69,1.26) | 0.672 |
| Reduced HDL-C | 1.02(0.78,1.34) | 0.861 |
| Elevated BP | 1.71(1.27,2.34) | <0.001 |
| Elevated glucose | 1.41(1.07,1.86) | 0.014 |

BMI, body mass index; HDL-C, high density lipoprotein cholesterol; SBP, systolic blood pressure; DBP, diastolic blood pressure; TG, triglycerides; BP, blood pressure.

**Table S5. Association between metabolic syndrome and perioperative ischemic stroke in sensitivity analyses.**

|  | **Perioperative ischemic stroke** |  |
| --- | --- | --- |
|  | **OR (95% CI) per unit incrementor for presence of categorical exposure** | ***P* value** |
| BMI | 1.02(0.98,1.06) | 0.265 |
| Triglycerides | 1.04(0.9,1.17) | 0.593 |
| HDL cholesterol | 0.99(0.64,1.2) | 0.956 |
| Glucose | 1.13(1.05,1.2) | <0.001 |
| SBP | 1.02(1.01,1.03) | <0.001 |
| DBP | 1.03(1.02,1.05) | <0.001 |
| MetS | 1.56(1.18,2.06) | 0.002 |
| Obesity | 1.06(0.63,1.68) | 0.817 |
| Elevated TG | 1.1(0.82,1.47) | 0.515 |
| Reduced HDL-C | 1.01(0.77,1.32) | 0.955 |
| Elevated bp | 1.96(1.4,2.79) | <0.001 |
| Elevated glucose | 1.52(1.15,2) | 0.003 |

BMI, body mass index; HDL-C, high density lipoprotein cholesterol; SBP, systolic blood pressure; DBP, diastolic blood pressure; TG, triglycerides; BP, blood pressure.

**Table S6. Correlation analysis among key perioperative factors and metabolic syndrome components.**

| **Coefficient** | **Glucose** | **BMI** | **Triglycerides** | **HDL-C** | **LDL-C** | **Preoperative MAP** | **Duration of Procedures** | **Age** | **Sex** | **ASA classification** | **MetS** |
| --- | --- | --- | --- | --- | --- | --- | --- | --- | --- | --- | --- |
| **Glucose** | 1 | 0.15 | 0.18 | -0.13 | 0.07 | 0.15 | 0.03 | 0.2 | -0.07 | 0.13 | 0.34 |
| **BMI** | 0.15 | 1 | 0.28 | -0.24 | 0.17 | 0.29 | 0 | 0.1 | -0.11 | 0.07 | 0.36 |
| **Triglycerides** | 0.18 | 0.28 | 1 | -0.39 | 0.14 | 0.15 | 0.01 | 0.06 | -0.1 | 0.04 | 0.45 |
| **HDL-C** | -0.13 | -0.24 | -0.39 | 1 | 0.09 | -0.07 | -0.07 | -0.02 | 0.31 | -0.06 | -0.37 |
| **LDL-C** | 0.07 | 0.17 | 0.14 | 0.09 | 1 | 0.13 | 0 | 0.17 | 0.05 | 0.04 | 0.08 |
| **Preoperative MAP** | 0.15 | 0.29 | 0.15 | -0.07 | 0.13 | 1 | 0.02 | 0.3 | -0.12 | 0.16 | 0.3 |
| **Duration of Procedures** | 0.03 | 0 | 0.01 | -0.07 | 0 | 0.02 | 1 | 0.04 | -0.08 | 0.08 | 0.03 |
| **Age** | 0.2 | 0.1 | 0.06 | -0.02 | 0.17 | 0.3 | 0.04 | 1 | -0.04 | 0.36 | 0.16 |
| **Sex** | -0.07 | -0.11 | -0.1 | 0.31 | 0.05 | -0.12 | -0.08 | -0.04 | 1 | -0.04 | 0.02 |
| **ASA Classification** | 0.13 | 0.07 | 0.04 | -0.06 | 0.04 | 0.16 | 0.08 | 0.36 | -0.04 | 1 | 0.13 |
| **MetS** | 0.34 | 0.36 | 0.45 | -0.37 | 0.08 | 0.3 | 0.03 | 0.16 | 0.02 | 0.13 | 1 |
|  |  |  |  |  |  |  |  |  |  |  |  |
| ***P*-value** | **Glucose** | **BMI** | **Triglycerides** | **HDL-C** | **LDL-C** | **Preoperative MAP** | **Duration of procedures** | **Age** | **Sex** | **ASA classification** | **MetS** |
| **Glucose** | 0 | 0 | 0 | 0 | 0 | 0 | 0 | 0 | 0 | 0 | 0 |
| **BMI** | 0 | 0 | 0 | 0 | 0 | 0 | 0.07 | 0 | 0 | 0 | 0 |
| **Triglycerides** | 0 | 0 | 0 | 0 | 0 | 0 | 0 | 0 | 0 | 0 | 0 |
| **HDL-C** | 0 | 0 | 0 | 0 | 0 | 0 | 0 | 0 | 0 | 0 | 0 |
| **LDL-C** | 0 | 0 | 0 | 0 | 0 | 0 | 0.17 | 0 | 0 | 0 | 0 |
| **Preoperative MAP** | 0 | 0 | 0 | 0 | 0 | 0 | 0 | 0 | 0 | 0 | 0 |
| **Duration of Procedures** | 0 | 0.07 | 0 | 0 | 0.17 | 0 | 0 | 0 | 0 | 0 | 0 |
| **Age** | 0 | 0 | 0 | 0 | 0 | 0 | 0 | 0 | 0 | 0 | 0 |
| **Sex** | 0 | 0 | 0 | 0 | 0 | 0 | 0 | 0 | 0 | 0 | 0 |
| **ASA Classification** | 0 | 0 | 0 | 0 | 0 | 0 | 0 | 0 | 0 | 0 | 0 |
| **MetS** | 0 | 0 | 0 | 0 | 0 | 0 | 0 | 0 | 0 | 0 | 0 |
